# Supplementary material for: Patterns and Functional Insights of DNA Methylation Variation in a South American Mayfly Across an Agriculturally Impacted Semi-Arid Watershed
Source: Biology (Basel). 2025 Dec 31;15(1):90. doi: 10.3390/biology15010090 (PMC12784713; doi:10.3390/biology15010090)
Supplement: Supplementary file 1 [file biology-15-00090-s001.zip › Supplementary_Material_F_20251126.pdf]

Supplementary Information

Title:
Patterns and functional insights of DNA methylation in a South American mayfly across an agriculturally impacted semi-arid watershed

Authors:
Angéline Bertin*, Ana María Notte, Bouziane Moumen, Diana Coral-Santacruz, Frédéric Grandjean, Nicolas Gouin

* Corresponding author, email: abertin@userena.cl

CONTENT

Tables:

Table S1. Information on sampling sites of *Andesiops torrens* populations and sample sizes used for methylRAD analyses.

Table S2. Sequencing depth and methylation levels of the 291 discriminating methylRAD loci identified in the population-based assignment analysis. Sheet 1 contains the sequencing depth of each locus and Sheet 2 their methylation levels for all individuals included in the study.

Table S3. Sequencing depth and methylation levels of the 112 discriminating methylRAD loci identified in the *de novo* grouping analysis. Sheet 1 contains the sequencing depth of each locus and Sheet 2 their methylation levels for all individuals included in the study.

Table S4. Genomic information of the most structuring methylRAD markers identified in the population-based analysis of *Andesiops torrens* and mapped to the draft genome. For each discriminant marker, the table indicates, among other variables, its genomic position, corresponding gene identifier, gene name, and the gene symbol used for Gene Ontology (GO) enrichment analysis.

Table S5. Gene Ontology (GO) enrichment results for hypermethylated markers identified across population clusters of *Andesiops torren*s.

Table S6. Genomic information of the most structuring methylRAD markers identified in the *de novo* analysis of *Andesiops torrens* and mapped to the draft genome. For each discriminant marker, the table indicates, among other variables, its genomic position, corresponding gene identifier, gene name, and the gene symbol used for Gene Ontology (GO) enrichment analysis.

Table S7. Gene Ontology (GO) enrichment results for hypermethylated markers identified across the *de novo* groups of *Andesiops torrens*.

Figures:

Figure S1. Breakpoint analyses identifying minimal informative marker sets for DAPC assignment.

Figure S2. Determination of the optimal number of de novo groups.

Figure S3. Genomic distribution of the discriminant markers identified in (a) the geographical-based DAPC analysis and (b) the *de novo* DAPC analysis.

Table S1. Information on sampling sites of *Andesiops torrens* populations and sample sizes used for methylRAD analyses (N). Site #: site codes follow Gouin et al. (2019, 2023) and correspond to those shown in Figure 1.

Site #	Site name	Catchment	Latitude	Longitude	Altitude (m)	N
1	Las Juntas	Río Grande	-30.717	-70.882	453	10
2	Garreton	Río Grande	-30.736	-70.869	504	9
3	Panguecillo	Río Grande	-30.781	-70.824	594	10
4	Peñon	Río Grande	-30.808	-70.807	638	9
5	Barrancones	Río Rapel	-30.748	-70.751	907	10
6	Semita	Río Grande	-30.826	-70.789	671	10
7	Gallardina	Río Grande	-30.844	-70.777	694	9
8	Sol Praderas	Río Rapel	-30.761	-70.708	1016	10
9	Pulpica	Río Grande	-30.883	-70.78	774	10
10	Vado Hondo	Río Grande	-30.907	-70.78	834	9
11	Mollacas	Río Rapel	-30.75	-70.647	1140	9
12	Pedregal	Río Mostazal	-30.849	-70.697	894	10
13	Cuyano	Río Grande	-30.928	-70.772	883	9
14	Molles1	Río Rapel	-30.745	-70.624	1195	10
15	Palomo	Río Rapel	-30.731	-70.61	1300	9
16	Maqui	Río Mostazal	-30.83	-70.662	1022	10
17	Molles2	Río Rapel	-30.742	-70.601	1276	10
18	Cisterna	Río Grande	-30.958	-70.768	938	10
19	Tulahuen1	Río Grande	-30.978	-70.743	1000	10
20	Maiten1	Río Mostazal	-30.814	-70.613	1242	9
21	Tulahuen2	Río Grande	-30.997	-70.725	1054	9
22	Maiten2	Río Mostazal	-30.8	-70.59	1402	9
23	Maiten3	Río Mostazal	-30.794	-70.58	1444	10
24	Tulahuen3	Río Grande	-31.011	-70.659	1237	9
25	Barrancas	Río Cogotí	-31.106	-70.907	949	9
26	Tulahuen4	Río Grande	-31.013	-70.633	1297	10
27	Ramadas1	Río Grande	-31.023	-70.594	1363	9
28	Durazno	Río Cogotí	-31.124	-70.846	1154	9
29	Ramadas2	Río Grande	-31.011	-70.579	1394	9
30	Turbio	Río Turbio	-31.038	-70.575	1497	10

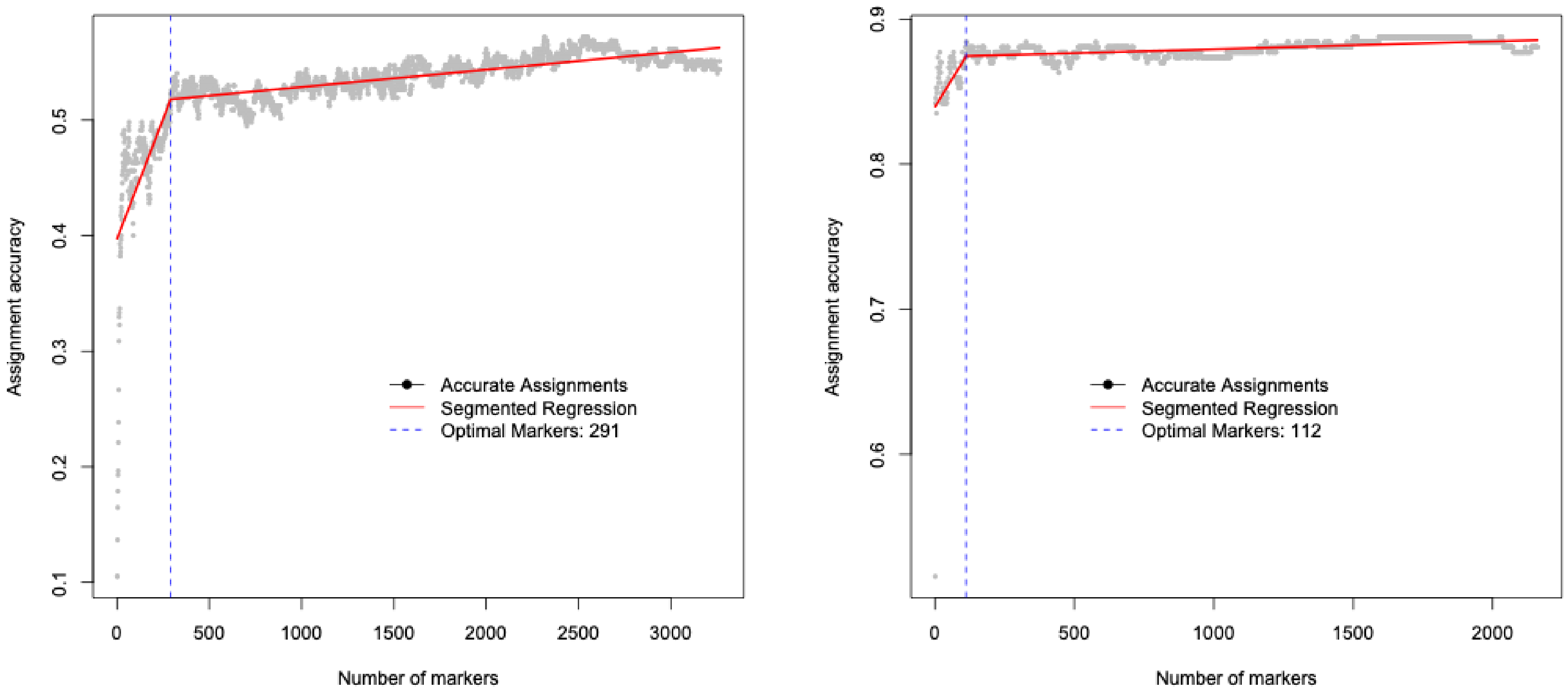

a b

Figure S1. Breakpoint analyses identifying minimal informative marker sets for DAPC assignment in (a) the geographic location-based analysis and (b) the de novo grouping analysis.

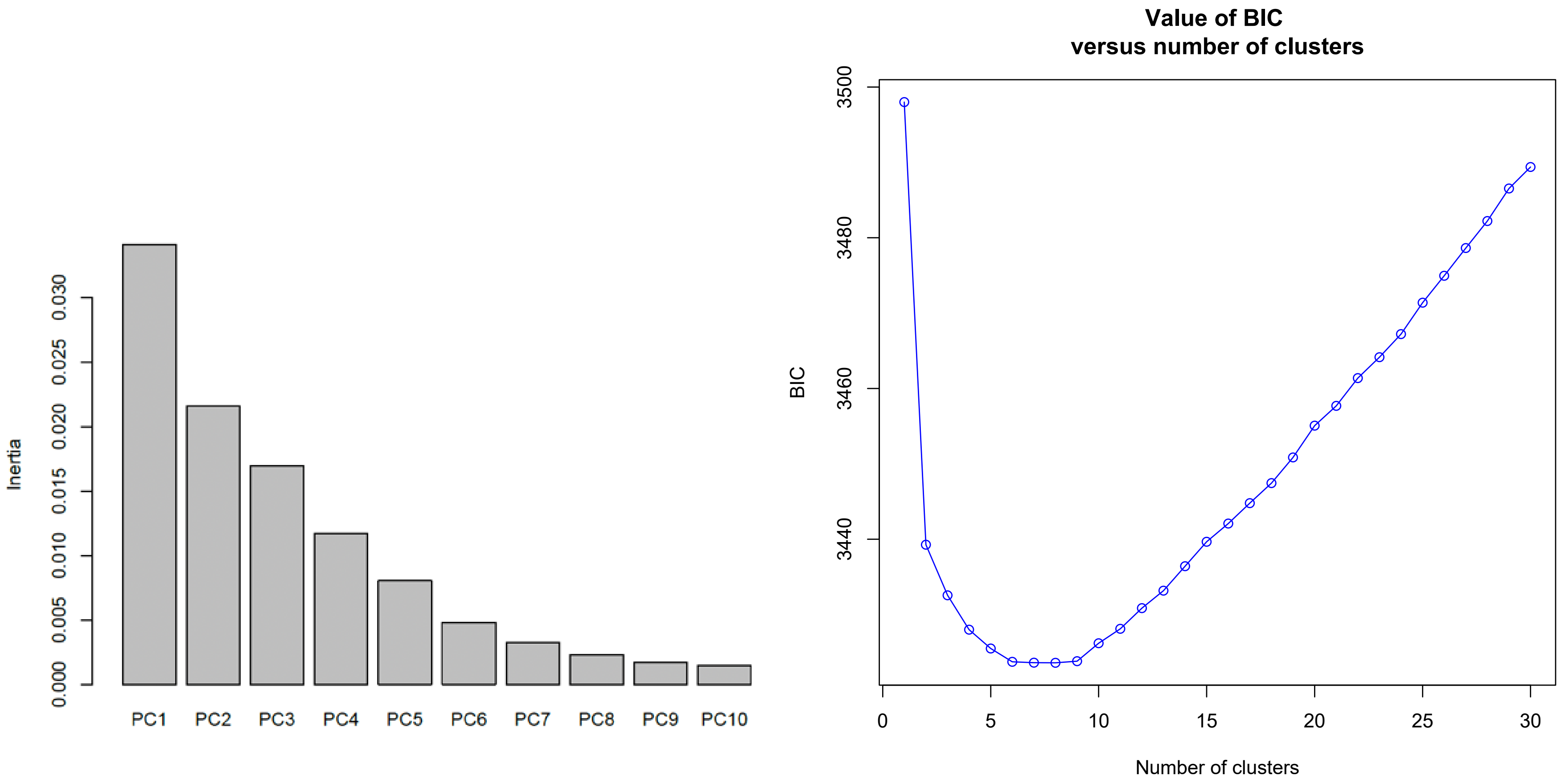

(a) (b)

Figure S2. Determination of the optimal number of de novo groups from methylation profiles of *Andesiops torrens* based on (a) inpection of the PCA cree plot and (b) Bayesian Information Criterion (BIC) values across K-means clustering solutions.

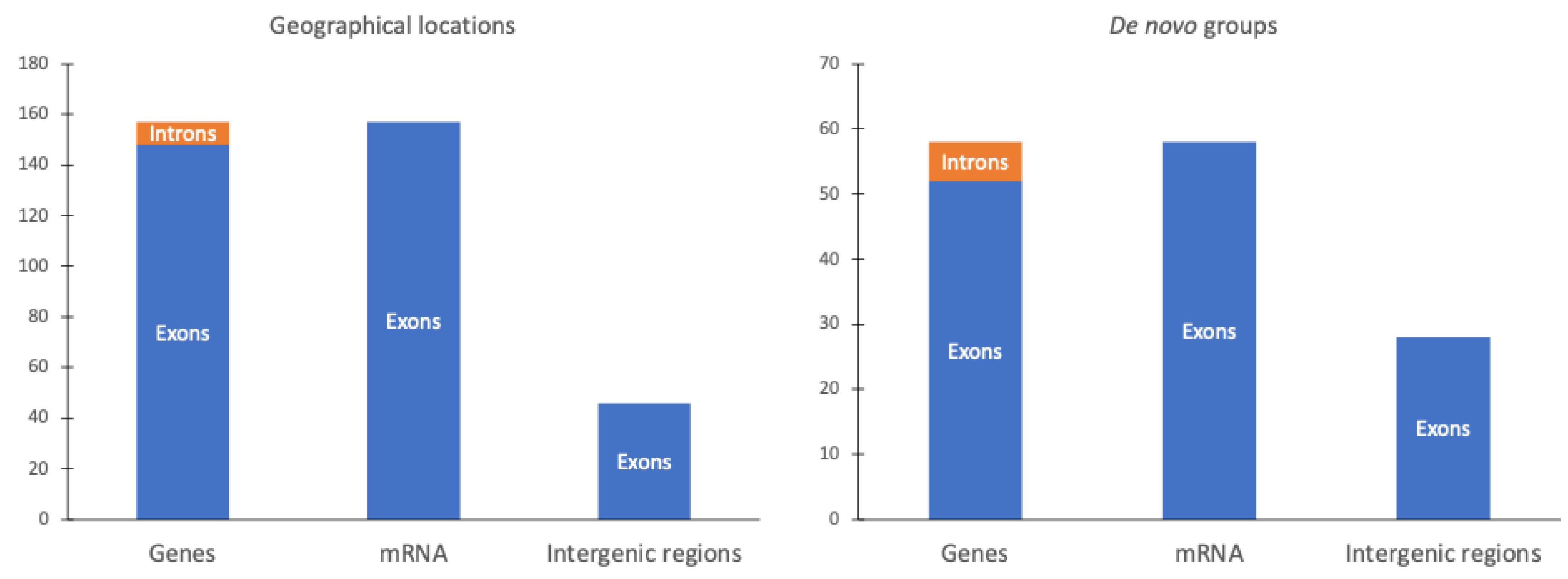

(a) **(b)**

Figure S3. Genomic distribution of the discriminant markers identified in (a) the geographical-based DAPC analysis and (b) the *de novo* DAPC analysis.
